# Current Innovations in Endoscopic Therapy for the Management of Colorectal Cancer: From Endoscopic Submucosal Dissection to Endoscopic Full-Thickness Resection 

**DOI:** 10.1155/2014/925058

**Published:** 2014-04-30

**Authors:** Shintaro Fujihara, Hirohito Mori, Hideki Kobara, Noriko Nishiyama, Tae Matsunaga, Maki Ayaki, Tatsuo Yachida, Asahiro Morishita, Kunihiko Izuishi, Tsutomu Masaki

**Affiliations:** ^1^Department of Gastroenterology and Neurology, Faculty of Medicine, Kagawa University, 1750-1 Ikenobe, Miki-cho, Kita-gun, Kagawa 761-0793, Japan; ^2^Department of Gastroenterological Surgery, KKR Takamatsu Hospital, Kagawa, Japan

## Abstract

Endoscopic submucosal dissection (ESD) is accepted as a minimally invasive treatment for colorectal cancer. However, due to technical difficulties and an increased rate of complications, ESD is not widely used in the colorectum. In some cases, endoscopic treatment alone is insufficient for disease control, and laparoscopic surgery is required. The combination of laparoscopic surgery and endoscopic resection represents a new frontier in cancer treatment. Recent developments in advanced polypectomy and minimally invasive surgical techniques will enable surgeons and endoscopists to challenge current practice in colorectal cancer treatment. Endoscopic full-thickness resection (EFTR) of the colon offers the potential to decrease the postoperative morbidity and mortality associated with segmental colectomy while enhancing the diagnostic yield compared to current endoscopic techniques. However, closure is necessary after EFTR and natural transluminal endoscopic surgery (NOTES). Innovative methods and new devices for EFTR and suturing are being developed and may potentially change traditional paradigms to achieve minimally invasive surgery for colorectal cancer. The present paper aims to discuss the complementary role of ESD and the future development of EFTR. We focus on the possibility of achieving EFTR using the ESD method and closing devices.

## 1. Introduction

Endoscopic submucosal dissection (ESD) is accepted as a minimally invasive treatment for early gastric cancer, but technical difficulties and an increased rate of complications have limited its use in the colorectum. ESD enables the en bloc resection of a specimen, but complications (mainly perforation and bleeding) are more frequently associated with ESD compared with endoscopic mucosal resection (EMR) [1, 2]. The balance between avoiding perforation and resecting a specimen sufficiently deep to permit accurate histopathological assessment is difficult to achieve. Due to the limitations of endoscopic techniques, a significant proportion of patients with large colonic polyps are referred for surgery [3].

In some cases, endoscopic treatment alone is insufficient for disease control, and laparoscopic surgery is required. The combination of laparoscopic surgery and endoscopic resection represents a new frontier in cancer treatment. Recent developments in advanced polypectomy and minimally invasive surgical techniques will enable surgeons and endoscopists to challenge current practice in colorectal cancer treatment.

In particular, endoscopic full-thickness resection (EFTR) of the colon might decrease the postoperative morbidity and mortality associated with segmental colectomy while enhancing the diagnostic yield compared to current endoscopic techniques. However, closure is necessary after EFTR and natural transluminal endoscopic surgery (NOTES). Endoscopic clips are often used for small mucosal defects but are not a suitable choice for large mucosal defects. Nonsurgical closure of the gastrointestinal wall using an over-the-scope clip (OTSC) (Ovesco, Tübingen, Germany) may be desired for inflammatory or neoplastic fistulae or iatrogenic perforations. Many endoscopic closure devices have undergone testing and evaluation in bench and animal models for use in minimally invasive surgical treatment and NOTES-associated procedures, including EFTR.

The present paper aims to discuss the complementary role of the ESD and the future development of EFTR. We focus on the possibility of achieving EFTR using the ESD method and closing devices.

## 2. ESD in the Colon

### 2.1. Indications for and Outcomes of Colorectal ESD

The specific indications for colorectal ESD, as recommended by the Colorectal ESD Standardization Implementation Working Group [4, 5], includes large-sized (>20 mm in diameter) lesions for which en bloc resection using snare EMR is difficult, including nongranular types of lateral spreading tumors (particularly those of the pseudodepressed type), lesions presenting type VI pit patterns, carcinoma with submucosal infiltration of less than 1000 microns, large depressed-type lesions, and large elevated lesions suspected to be carcinoma. Additional indications for ESD include mucosal lesions with fibrosis related to biopsy; sporadic tumors in chronic inflammation, such as in ulcerative colitis; and local residual carcinoma after endoscopic resection that fulfills the aforementioned criteria. An additional, often-cited indication for ESD is adenoma displaying a nonlifting sign.

ESD is not as widely performed in the colorectum compared with gastric ESD, even in Japan, because of the greater technical difficulty due to the anatomical features of the colon, including its longer length, narrower lumen, extensive flexion, and thinner walls, which increase the duration of the procedure and the risk of perforation [2, 4, 6–11].

Colorectal ESD remains more technically challenging than esophageal and gastric ESD for the following reasons. First, the anatomical features of the large intestine, including a long luminal organ with many folds and flexions, mean that the endoscope cannot be easily manipulated to reach certain lesions. Additionally, the intestinal wall is thin and easy to perforate. Importantly, the longer operation time increases the amount of air in the abdomen, causing greater paradoxical movement of the endoscope. The paradoxical movement of the endoscope during ESD due to the winding nature of the colorectum causes coagulation in the muscularis propria. This situation specifically occurs in the case of tumors located in the cecum, up to the descending colon. Second, peritonitis may occur if stool in the large intestine leaks through a perforation and into the abdominal cavity [12].

Table 1 summarizes the outcomes of colorectal ESD using previous reports from single-institution studies [7, 13–31]. The outcomes of colorectal ESD, as summarized from previous reports from multicenter studies, reveal rates of en bloc resection (endoscopic) and complete en bloc resection (histological) of 88.8% and 83.8%, respectively [2, 5]. In another study, Farhat et al. [32] reported en bloc resection (endoscopic) and complete en bloc resection (histological) rates of 67.1% and 62.4%, respectively. The perforation rate was 3.3–14.0%, and the delayed perforation rate was 0.4–0.7%. Additionally, postoperative bleeding occurred in 1.5–2.1% of cases [12].

The high rate of perforation is due to the thinness of the colorectal wall compared to the gastric wall. A few clinical studies have focused on the risk factors that predict perforation during ESD for colorectal tumors. Large lesions, fibrosis, colonic location (due to a thinner wall than in the rectum), and less experience performing ESDs might be risk factors for perforation during ESD [2, 7, 33, 34].

**Figure 1:**
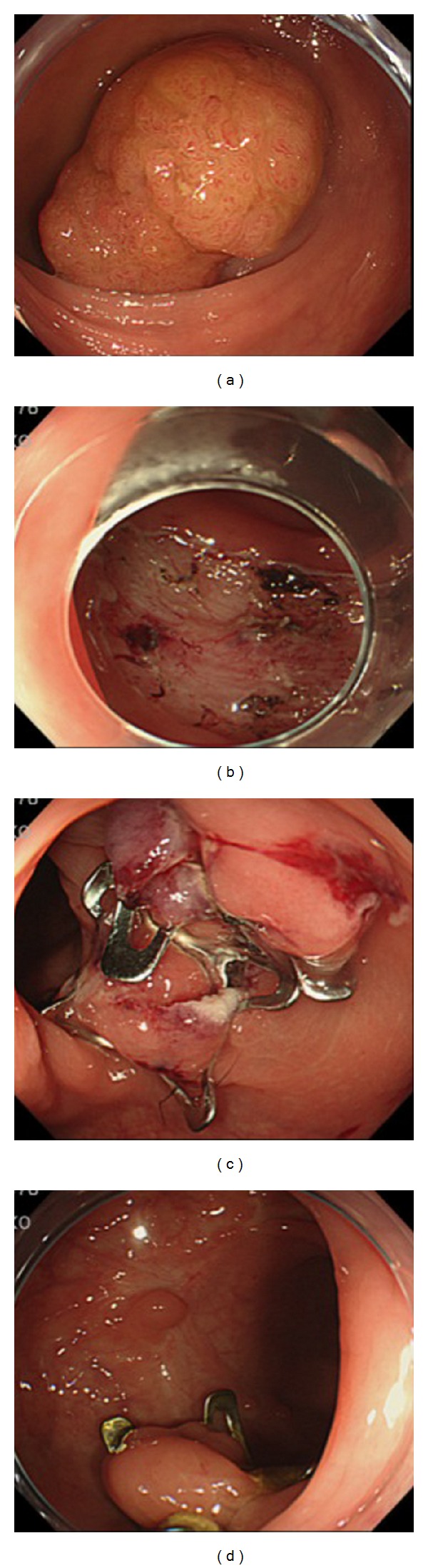
Endoscopic closure of an artificial ulcer with conventional clips and an OTSC system. (a) A large tumor, measuring 55 mm in diameter, located in the upper rectum. (b) A large mucosal defect after colorectal ESD. (c) Complete closure was performed using an OTSC system. (d) The endoscopic view at postoperative day 333.

Closure of mucosal defects created during ESD is not routinely practiced in Japan, although some experts believe that such closure may decrease the risk of delayed bleeding and possibly perforation. At present, closure of the typically large ESD mucosal defect is impractical and technically challenging with currently available devices (e.g., hemoclips). The rate of perforation is higher than the rate of postoperative bleeding, but methods for preventing severe complications remain to be established for the management of postcolorectal ESD. Prophylactic closure with hemoclips may be effective in preventing the postoperative bleeding related to the endoscopic mucosal resection (EMR) of large (≥2 cm) sessile or flat colorectal lesions. In a cohort of polyps for which all polypectomies were performed using pure low-power coagulation current, the risk of delayed postpolypectomy hemorrhage decreased from 9.7% to 1.8% when comparing polypectomy sites that were not clipped with those that were completely clipped closed [35]. However, prophylactic clip closure has no benefit for the prevention of complications (postpolypectomy syndrome and perforation) other than delayed hemorrhage. We have reported that the prophylactic closure of large mucosal defects after colorectal ESD reduces the inflammatory reaction and relieves patient symptoms after colorectal ESD [36] (Figure 1). However, we could not obtain sufficient evidence to determine whether the prophylactic closure prevented delayed perforation and postoperative bleeding because of the small sample size and retrospective nature of the study. Future studies will require a larger sample size and a prospective study design.

At the completion of the resection, the artificial ulcer is carefully inspected for any visible vessels. These are typically coagulated using the coag grasper to prevent delayed bleeding. This is particularly important and may explain the observation that ESD has a generally lower rate of delayed bleeding compared with EMR [9].

### 2.2. Various Knives and Colorectal ESD

A large number of devices for ESD are now available in Japan [7, 13–31] (Table 1). These devices are divided into two broad categories: the needle-knife type and the grasping (scissors) type. The needle-knife type can be further subdivided into the uncovered and covered (insulated) types. Among the obtuse, short-tipped types are the FlushKnife (Fujifilm Medical, Tokyo, Japan), the DualKnife (Olympus Medical Systems Co., Tokyo, Japan), the B-Knife (Zeon Medical, Tokyo, Japan), and the Splashneedle (Pentax Co., Tokyo, Japan) [33, 37, 38]. The FlushKnife and the Splashneedle are capable of injecting substances into the submucosa. These devices eliminate the need to switch between the knife and the injection needle [37, 39]. The DualKnife has a ball disk at the tip of the knife, allowing the user to hook the submucosa. The B-Knife is the only bipolar knife; burning of the muscularis propria layer is reduced with this knife compared with other monopolar knives. For this reason, the B-Knife may produce fewer complications, particularly perforations. Adapting to another type of knife requires time and practice by the user.

The HookKnife (Olympus Medical Systems Co., Tokyo, Japan) is particularly useful when dissection of the submucosa is difficult due to poor elevation of the submucosa [40].

Obtuse short-tipped knives, such as the DualKnife and the FlushKnife, can easily cause perforation when used on the thin colon wall due to the presence of folds. By contrast, it is difficult to cause perforations while using the HookKnife because the submucosa can be hooked and separated from the muscularis propria and cut safely [41]. However, use of the HookKnife requires an assistant to rotate the direction of the knife for proper device efficacy.

The insulated-tipped (IT) knife 2 (Olympus Medical Systems Co., Tokyo, Japan), the efficacy of which has been reported to be satisfactory in ESD for gastric tumors, is used in certain institutions [1]. The long blade of the ITknife 2 enables rapid dissection, thereby shortening the operation time and enabling coagulation in small vessels. However, mastering the ITknife 2 is difficult because the angle of the ITknife 2 to the mucosa is critical. In addition, the long blade of the ITknife 2 may cause large perforations. In Japan, a new type of insulated-tipped knife (called the ITknife nano (Olympus Optical Co., Ltd, Tokyo, Japan)) is used; this knife is smaller than the ITknife 2 and was invented to improve the safety and speed of submucosal dissection in the colon. A grasping-type scissor forceps has also been reported as another novel knife [42].

The Clutch Cutter device (Fujifilm, Tokyo, Japan) and SB knife Jr (Sumitomo Bakelite) are the major types of grasping-type scissor forceps [31, 43].

Cutting methods that involve the use of grasping-type scissors forceps, without fixing the knife to the target, are associated with a potential risk of major complications, such as perforation and bleeding, due to unexpected incision (i.e., because of cardiac or respiratory movement) [43]. In our institution, the DualKnife is primarily used, while the grasping scissors forceps is used when the risk of perforation is high due to the poor elevation of the submucosa and when facing the colon wall.

## 3. EFTR in the Colon

### 3.1. Indications for EFTR

The indications for EFTR include adenoma, intramucosal carcinomas, and slight submucosal invasion (<1000 micrometers below the muscularis mucosa; sm1) without lymphovascular infiltration, which are similar to the indications for ESD.

The risk of local recurrence is significantly higher in high-risk patients with submucosal rectal cancer than in patients with submucosal colon cancer who are treated with only endoscopic resection [44]. In these cases, hybrid NOTES or laparoscopic and endoscopic cooperative surgery might be recommended to perform lymphadenectomy. However, before resection of the tumor, we were unable to identify nodal involvement. Several reports have described the supplementation of ESD with sentinel node biopsy by NOTES [45, 46].

**Figure 2:**
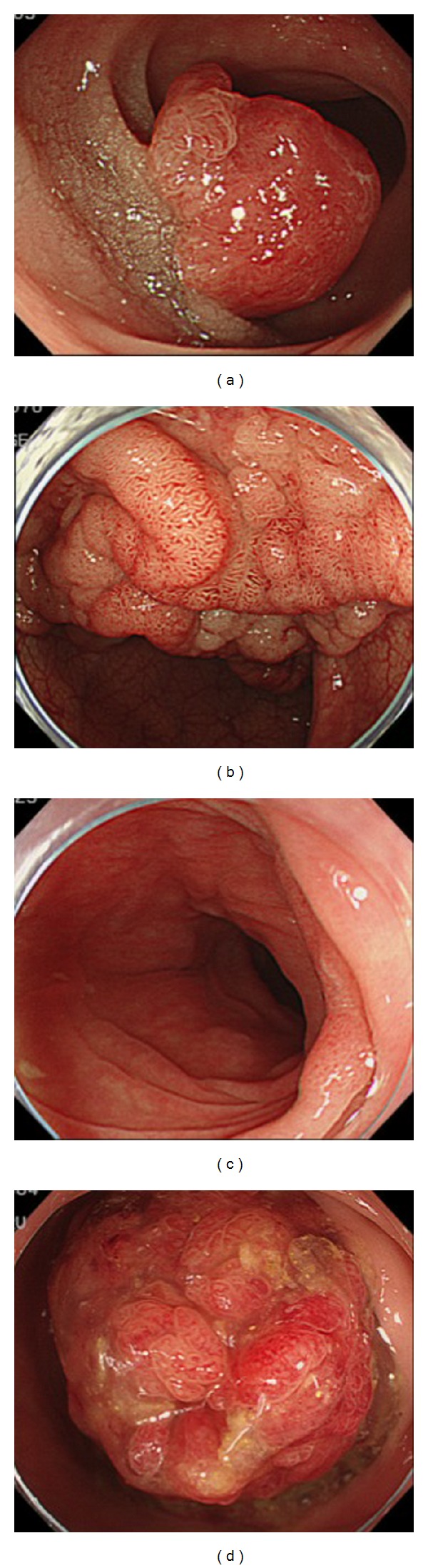
Difficult lesions with endoscopic treatment. (a) Deeper invasion of the submucosa in colorectal cancer. (b) A laterally spreading tumor occupying more than one-third of the bowel circumference or spanning more than two haustral folds. (c) Remnant lesion. (d) A large pedunculated polyp.

Additionally, the indications may include the following: (1) technically difficult ESD for early colorectal cancer (Figure 2) and (2) carcinoid tumors. These two aspects are considered to determine the need for EFTR for each lesion. Performing colorectal ESD is challenging in the presence of technically difficult lesions with severe fibrosis; recurrent lesions; lesions located at the bottom of the cecum, near the terminal ileum, and in the appendix; and large pedunculated polyps.

Isomoto et al. [13] reported that right-side colonic location and fibrosis were the most significant contributors to incomplete resection and that perforation was associated with large tumor size (30 mm) and the presence of fibrosis. They also reported that the risks of incomplete resection and perforation increased substantially when the contributive factors for each were combined. Matsumoto et al. reported that in cases of lesions with severe fibrosis, the rate of complete en bloc resection was low, and the perforation rate was high, even when ESD was performed by an experienced operator. However, clarification of the presence and extent of fibrosis before actual colorectal ESD is obviously impossible [14]. In contrast, Yoshida et al. reported that knife coagulation was the most common cause of perforation [47].

Choi et al. reported that in cases of a giant pedunculated polyp with (1) poor visualization of the stalk, (2) technical difficulties in snare positioning for en bloc resection, or (3) the need for trimming of the head, piecemeal snare polypectomy was not attempted, and endoscopic submucosal dissection was performed instead [48]. For large colonic polyps occupying more than one-third of the bowel circumference or spanning more than two haustral folds, combined endolaparoscopic resection is a viable treatment modality [4, 10, 11]. Recurrent lesions with fibrosis and large pedunculated polyps can be indications for EFTR, considering the longer operation time and the risk of complication, if they fulfill the criterion of no nodal metastasis.

Endoscopic treatment is considered to be curative for small carcinoid tumors (<10 mm) with an extremely low risk of metastasis [49]. Recently, ESD was reported to be an effective method for the treatment of rectal carcinoid tumors [50–52]. When the lesions are intermediate in size or massively invade the submucosal layer, which may result in tumor-positive margin resection, these can be indications for ESD or EFTR.

### 3.2. Procedural Methods

Schurr et al. [53] and Rajan et al. [54] reported the use of a full-thickness resection device (FTRD) consisting of a hollow, flexible shaft with a resection head and tissue manipulators. In a study by Schurr et al. [53], the FTRD was under development, and a conventional circular surgical stapler was used as a predicate device to imitate the procedure. Two studies reported in the same paper and an additional study published by Rajan et al. [54] were conducted using the FTRD with the goal of assessing the feasibility and safety of EFTR using this novel device. The tissue fold was excised, resulting in a stapled anastomosis. Use of the FTRD was focused on flat, elevated lesions with a diameter of up to 3 cm [53], including both adenomas and early colorectal carcinomas. Compared to TEM, the advantage of FTRD is that it can be used to target both rectal and colonic lesions up to the splenic flexure [53]. However, FTRD is disadvantageous for lesions involving flexures and curves because it requires straightening of the colon.

Raju et al. [55] reported on circular, full-thickness resection of the colon at approximately 20 cm from the anus (peritoneal portion of the colon) with a band-ligation device. They performed closure by using a median of 7 clips (range, 6–13 clips) and produced a median length of the closed defect on the serosal side of 3 cm (range, 1.5–4 cm).

Von Rentlen et al. [56, 57] and Rieder et al. [58] described several variations of a grasp-and-snare technique. Von Renteln et al. [56] performed both pre- and postresection closure using an OTSC (Ovesco Endoscopy, Tubingen, Germany). A tissue anchor (Ovesco, Endoscopy) was inserted through a double-channel gastroscope (2T160, Olympus, Hamburg, Germany) to grasp the bowel wall and create a pseudopolyp. The base of the pseudopolyp was ligated with an Endoloop (HX-400U-30, Olympus) before snare resection.

A disadvantage of the grasp-and-snare technique is that it makes it difficult to produce a specimen with an adequate horizontal clearance margin. Rieder et al. [58] described a preresection closure method with laparoscopic overview using an OTSC mounted on a dual-channel gastroscope (GIF-2T-160, Olympus). Kennedy et al. [59] described a laparoendoscopic procedure using two endoscopes (R-scope, Olympus Keymed, and GIF-Q240, Olympus Keymed). Circumferential APC marks were placed at 1 cm from the edge of a simulated polyp created by the submucosal injection of ink. Prototype BraceBars (Olympus Medical Systems, Olympus), which are similar to the TAS system, were placed laparoscopically to invert the area bearing the polyp. Three pairs of BraceBars placed 1 cm from the endoluminal APC marks were required to invert the area, and the inversion site was oversewn laparoscopically. The created endoluminal fold was retracted with one endoscope (GIF-Q240, Olympus Keymed), and excision was performed using an endoscopic knife inserted through a working endoscope (R-scope, Olympus Keymed). Recently, Picasso et al. reported resection of flat, elevated polyps (20 mm) using EMR and full-thickness closure with an OTSC device in humans [60].

EFTR procedural methods are summarized in Table 2. These methods (1) mostly involve the use of a dual-channel scope, (2) require a laparoscopic assist, (3) are mainly used to resect the inner wall of the main lesion with a snare, and (4) produce better results for a small defect of the colorectal wall than with a large defect because of the need to close the mucosal defect completely.

The advantage of these methods is the ability to easily and quickly resect the main lesion and quickly close the colon wall defect. These methods are also favorable for preventing peritoneal infection and the dissemination of cancer cells to the peritoneal cavity. However, a dual-channel scope cannot easily turn around in a small circle compared to single-channel scopes. Thus, performing a complicated endoscopic treatment using a dual-channel scope may be difficult. The larger size of flat, elevated lesions (e.g., lateral spreading tumors) is difficult to resect when using only a snare. Endoscopic treatment for larger-sized lesions requires additional devices to support closure and suturing.

### 3.3. The Potential for and Limitations of EFTR Using ESD Devices

Recently, endoscopic resection of gastric intestinal stromal tumors (GISTs) or submucosal tumors has been performed with the use of ESD techniques and laparoscopic-assisted suturing, or so-called laparoscopic and endoscopic cooperative surgery (LECS) [61] and hybrid NOTES [62]. When a large defect is created in the colon, it is difficult to maintain clear vision using the endoscopic platform for resection on the colon wall because of the air leaking out into the peritoneal cavity. To address the problem of air leaking out and collapsing the colon wall, we attempted EFTR with laparoscopic assistance for GISTs by using the equally spaced perforation technique, which prevents air from leaking out into the peritoneal cavity [62].

**Figure 3:**
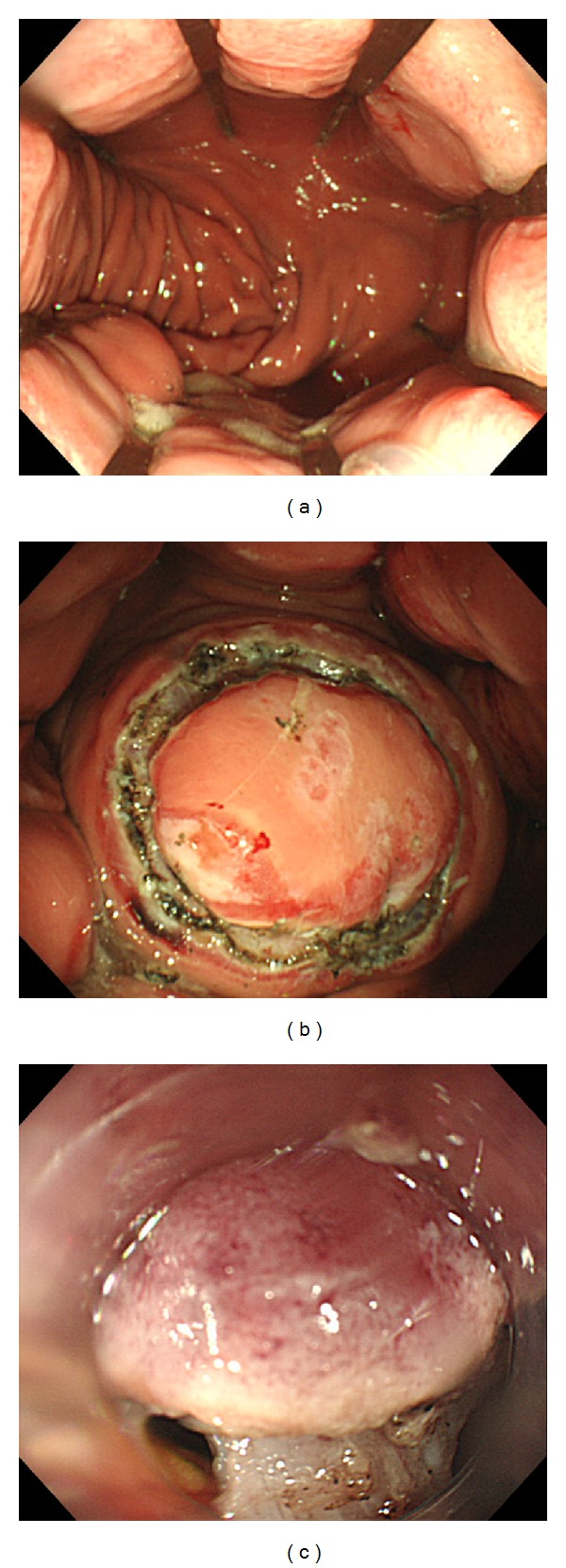
EFTR using ESD devices. (a) The mucosa was cut circumferentially, and the submucosal layer was then cut. (b) Next, we created a small hole in the muscular layer using a needle-type knife. (c) Equidistant small dots enabled the correct resection of the muscular layer using the ITknife.

The equally spaced perforation technique is a resection method for easily achieving EFTR in which 1.5 mm perforation holes are created at 10 mm intervals and used as landmarks to perform EFTR. The mucosa was first cut circumferentially, and the submucosal layer was then cut (Figure 3(a)). Next, we created a small hole in the muscular layer using a needle-type knife (Figure 3(b)). Equidistant small dots enabled the correct resection of the muscular layer using the ITknife (Figure 3(c)). Massive and spurting bleeding occasionally occurred during dissection of the muscular layer. Hiki recommends that the ESD procedure be used for submucosal, but not seromuscular, dissection because of poor bleeding control during treatment of the muscular layer [61]. For that reason, laparoscopic overview and assistance during EFTR improve the safety of the procedure by creating traction and laparoscopic hemostasis.

Several EFTR procedures have been described, and several problems existed when EFTR was performed using an ESD device. Postmortem findings from animal survival studies suggest a risk of peritoneal contamination and infection. Additionally, postresection closure risks the seeding of malignant cells into the peritoneal cavity in the case of early colorectal cancer.

## 4. Endoscopic Suturing Devices

### 4.1. OTSC

An OTSC (Ovesco, Tübingen, Germany) has been developed for the closure of small mural defects and bleeding ulcers [63]. Several case series have demonstrated the successful use of the OTSC in the closure of acute GI perforations, anastomotic leaks, bleeding lesions, and chronic GI fistulae [63–71].

Kirschniak et al. [63] initially described the use of this device in the successful closure of 2 small (4 and 8 mm) iatrogenic colonic perforations and 2 deep mucosal resection sites (also 4 and 8 mm in size) in the colonic and gastric walls. The advantage of the OTSC is that it can easily and rapidly completely close a mural defect compared to other devices.

We evaluated the efficacy and safety of the OTSC in OTSC placement for 23 patients with GI bleeds, perforations, GI fistulae, and post-ESD artificial ulcers. The overall success rate was 82.6%. A large lesion size (greater than 20 mm) and delayed diagnosis were the major contributing factors to unsuccessful cases [71]. OTSCs may have limited efficacy in the following situations: (1) larger mural defects, (2) chronic inflammation, and (3) repeated attempts at closure.

Von Rentlen et al. [56, 57] described ligation of the base of a pseudopolyp with an Endoloop (HX-400U-30, Olympus) and an OTSC loaded onto a cap at the end of the endoscope; the OTSC was applied at the base of the Endoloop before snare resection. Earlier studies evaluating OTSCs for the closure of defects were only performed for defects up to 18 mm in size [72, 73].

These 18 mm openings were created using balloon dilatation, facilitating tissue approximation compared to EFTR defects. Outcomes studies have demonstrated that EFTR defects up to 2.7 cm can be adequately closed using OTSCs in the majority of cases. For defects larger than 2.7 cm, OTSCs are not sufficient. For some colonic defects >2.7 cm, serial OTSC placement can be used with success, but closure may be associated with luminal obstruction depending on the location and configuration of the initial resection. Furthermore, large EFTR defects induce a distinct luminal collapse, rendering endoscopic closure very difficult [57].

We previously reported successful use of the OTSC in GI bleeds, perforations, and fistulae. In our experience, large lesion size (greater than 20 cm) and delayed perforation were the major contributing factors in unsuccessful cases [71].

A potential indication for the removal of an OTSC could be its misplacement.

In our experience, it is difficult to deploy a second clip next to the first one. Several methods for removing OTSCs have been reported [74–76]. Removal of the first clip enables correct deployment of the second clip [77]. Arezzo et al. reported an interesting new technique involving the use of cold saline solution for the gentle removal of the OTSC [75]. Baron et al. described the use of argon plasma coagulation at high power; the optimal site for cutting the device appears to be at the hinge [76]. More recently, Fähndrich et al. described the use of an yttrium aluminum garnet laser to destroy and remove an OTSC* in vivo* [78].

However, regardless of the method used, the destroyed OTSC should be retrieved through an overtube to avoid tissue damage while retracting the OTSC through the gastroesophageal junction.

### 4.2. Overstitch Endoscopic Suturing System and Double-Arm Bar Suturing System

The Overstitch Endoscopic Suturing System (Apollo Endosurgery, Austin, Tex) is a disposable, single-use suturing device that is mounted onto a double-channel therapeutic endoscope and allows for the placement of either running or interrupted full-thickness sutures. The device represents an evolution of the previously described Eagle Claw device [79, 80].

This suturing device has been used successfully for the closure of persistent gastrocutaneous fistulae [81–86], fixation of esophageal stents, suturing of ulcers, and other indications. Kantsevoy et al. reported that closure of a large post-ESD mucosal defect in the colon using the Overstitch Endoscopic Suturing Device decreased treatment cost by eliminating the need for hospitalization.

**Figure 4:**
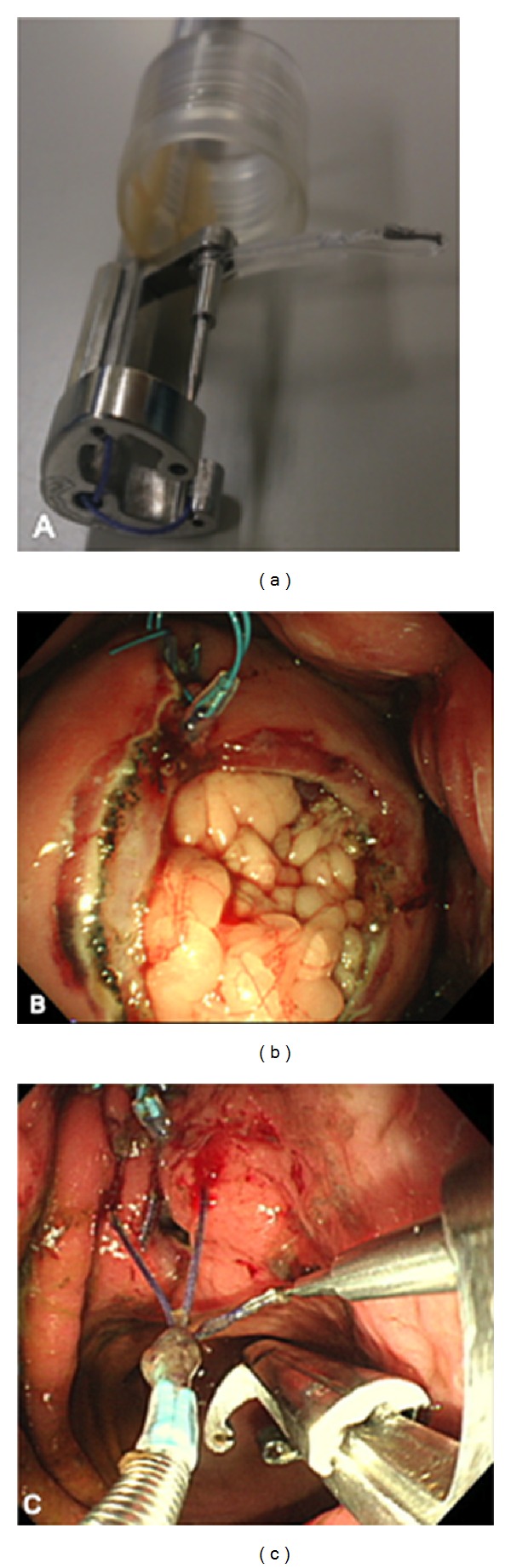
(a) Double-armed bar suturing system (DBSS). (b) Endoscopic view by using mechanical counter traction device. (c) Endoscopic suturing in animal experimental model.

Recently, we were challenged to perform an EFTR using the ESD method and to suture a large mucosal defect using a new suturing system and mechanical counter traction device in animal models [87–89] (Figure 4). For the suturing of resection wounds >40 mm in the stomach, these devices enabled the complete suture of a large mural defect. We aim to improve these devices so that large mural defects can be sutured more simply and quickly.

## 5. Conclusions

ESD has been established as a minimally invasive endoscopic treatment for tumors of the digestive tract wall and yields high en bloc resection rates for larger tumors of the esophagus, stomach, and colon. The establishment of EFTR and endoscopic suturing may cause the following problems: the dissemination of malignant cells, infection, hemostasis, and anastomotic leakage. Laparoscopic overview and assistance during EFTR improve the safety of the procedure by creating traction and laparoscopic hemostasis. Additionally, combining endoscopy and laparoscopy is important when endoscopic treatment alone is inadequate for disease control. Furthermore, innovative methods and new devices for EFTR and suturing will enable the local resection of tumors via minimally invasive surgery.

Innovative methods and new devices for EFTR and suturing are evolving and may change traditional paradigms to achieve minimally invasive surgery for colorectal cancer.

## Figures and Tables

**Table 1:** Summary of outcomes of colorectal ESD using previous reports from single institution studies.

Author	Year	Country	Number of cases	En bloc resection rate	Complete en bloc resection rate	Main device	Generator	Complication	
								Perforation rate	Post-ESD bleeding rate
Tamegai et al. [16]	2007	Japan	71	98.6%	95.8%	HookKnife	—	—	1.4%
Hurlstone et al. [17]	2007	UK	42	78.6%	73.8%	Flex knife, IT knife	—	2.4%	9.5%
Fujishiro et al. [7]	2007	Japan	200	91.5%	70.5%	Flex knife, HookKnife, electrosurgical knife	ICC-200 or VIO300D	6.0%	0.5%
Zhou et al. [18]	2009	China	74	93.2%	89.2%	Needle-knife, IT knife, hook knife	ICC-200	8.1%	1.4%
Isomoto et al. [13]	2009	Japan	292	90.1%	79.8%	Flex knife, flash knife, HookKnife	ICC-200 or VIO300D	7.9%	0.7%
Saito et al. [19]	2009	Japan	405	86.9%	—	Bipolar needle knife (B-knife), IT knife	—	3.5%	1.0%
Iizuka et al. [20]	2009	Japan	38	60.5%	57.9%	Flex knife	ICC200 or VIO300D	7.9%	—
Hotta et al. [21]	2010	Japan	120	93.3%	51.0%	Flex knife, flush knife, HookKnife	ICC200 or VIO300D	7.5%	—
Niimi et al. [22]	2010	Japan	310	90.3%	74.5%	Flex knife, HookKnife, electrosurgical knife	ICC200 or VIO300D	4.8%	1.6%
Yoshida et al. [23]	2010	Japan	250	86.8%	81.2%	FlushKnife	VIO300D	6.0%	2.4%
Toyonaga et al. [24]	2010	Japan	512	98.2%		Flex knife, FlushKnife	—	1.8%	1.6%
Matsumoto et al. [14]	2010	Japan	203	—	85.7%	Flex knife, HookKnife, DualKnife	—	6.9%	—
Uraoka et al. [25]	2011	Japan	202	91.6%	—	B-Knife, DualKnife, IT knife, mucosectome	—	2.5%	0.5%
Shono et al. [26]	2011	Japan	137	89.1%	85.4%	FlushKnife, HookKnife, precutting knife	—	3.6%	3.6%
Kim et al. [27]	2011	Korea	108	—	78.7%	Flex knife, HookKnife	VIO300D	20.4%	—
Lee et al. [28]	2011	Korea	499	95.0%	—	Flex knife, HookKnife	VIO300	7.4%	—
Probst et al. [29]	2012	Germany	76	81.6%	69.7%	HookKnife, IT knife, triangle knife	VIO300D	1.3%	7.9%
Okamoto et al. [30]	2013	Japan	30	100.0%	—	DualKnife, mucosectome-2 short blade	VIO300D	0.0%	0.0%
Nawata et al. [31]	2014	Japan	150	98.7%	97.3%	SB knife JR, IT knife nano	—	0.0%	0.0%

**Table 2:** Summary of endoscopic full-thickness resection (EFTR) procedures in an animal model.

Author	Year	Country	No. animals	Approach	Resection devices	Closure methods	Device	Procedure completed	Size of specimen (cm)	Procedure time (min)	Inoperative complication rate
Schurr et al. [53]	2001	USA	25	Endoscopy and laparotomy with endoscopic resection	Endoscopic FTRD	Pre-resection closure method	FTRD	100%	—	—	12%
Rajan et al. [54]	2001	USA	8	Endoscopic only	Endoscopic FTRD	Pre-resection closure method	FTRD	100%	Mean 3.6	Mean 30.2	50%
Raju et al. [55]	2009	USA	20	Endoscopic only	Endoscopic knife and snare	Post-resection closure method	T-tag	95%	Median 1.7	Median 50	0%
von Renteln et al. [56]	2010	Germany, Canada	8	Endoscopic only	Endoscopic snare	Pre-resection closure method	Endoloop and OTSC	100%	Mean 1.8	Mean 31.5	25%
			20		Endoscopic snare	Post-resection closure method	Twin-grasper and OTSC	45%	Mean 3.3	Mean 14.8	67%
Rieder et al. [58]	2011	Germany	2	Laparoscopically monitored endoscopic resection	Endoscopic snare	Pre-resection closure method	T-tag and OTSC	100%	Mean 2.2	Mean 33	0%
von Renteln et al. [57]	2011	Germany	8	Endoscopic only	Endoscopic snare	Pre-resection closure method	OTSC using a grasper	88%	7.6 cm^2^	Median 3	25%

FTRD: full-thickness device; OTSC: Over-The-Scope.
